# Biomechanical Identification of High-Risk Patients Requiring Permanent Pacemaker After Transcatheter Aortic Valve Replacement

**DOI:** 10.3389/fbioe.2021.615090

**Published:** 2021-07-09

**Authors:** Guangming Zhang, Rong Liu, Min Pu, Xiaobo Zhou

**Affiliations:** ^1^School of Biomedical Informatics, The University of Texas Health Science Center at Houston, Houston, TX, United States; ^2^Department of Internal Medicine/Cardiology, Wake Forest University School of Medicine, Winston-Salem, NC, United States

**Keywords:** transcatheter aortic valve replacement, atrioventricular block, calcification, stress, finite element method

## Abstract

**Background:**

Cardiac conduction disturbance requiring new permanent pacemaker implantation (PPI) is an important complication of TAVR that has been associated with increased mortality. It is extremely challenging to optimize the valve size alone to prevent a complete atrioventricular block (AVB).

**Methods:**

In this study, we randomly took 48 patients who underwent TAVR and had been followed for at least 2 years to assess the risk of AVB. CT images of 48 patients with TAVR were analyzed using three-dimensional (3D) anatomical models of the aortic valve apparatus. The stresses were formulated according to loading force and tissue properties. Support vector regression (SVR) was used to model the relationship between AVB risk and biomechanical stresses. To avoid AVB, overlapping regions on the prosthetic valve where AV bundle passes will be removed as cylindrical sector with the angle θ. Thus, the optimization of the valve shape will be predicted with the joint optimization of the θ and valve size R.

**Results:**

The average AVB risk prediction accuracy was 83.33% in the range from 0.8–0.85 with 95% CI for all cases; specifically, 85.71% for Group A (no AVB), and 80.0% for Group B (undergoing AVB after the TAVR).

**Conclusions:**

This model can estimate the optimal valve size and shape to avoid the risk of AVB after TAVR. This optimization may eliminate the excessive stresses to keep the normal function of both AV bundle and valve leaflets, leading to a favorable clinical outcome. The combination of biomechanical properties and machine learning method substantially improved prediction of surgical results.

## Introduction

Transcatheter Aortic Valve Replacement (TAVR) (Agnihotri et al., 2012) is a minimally invasive procedure to treat symptomatic patients with calcific aortic stenosis (AS). As an alternative to surgical aortic valve replacement for intermediate and high-risk patients with severe AS, TAVR has many advantages. Favorable outcomes require proper patient selection and meticulous procedural technique to avoid aortic insufficiency. One important complication of TAVR is the cardiac conduction disturbance which requires permanent pacemaker implantation (PPI) and has been associated with increased mortality. Without the help of pacemaker, atrioventricular block (AVB) as a clinical outcome of TAVR may result in sudden death. In clinical practice, aortic valve stenosis is usually diagnosed by cardiac CT and CT angiography (CTA). Based on the severity of aortic stenosis on CTs, the clinicians make decisions whether a patient needs TAVR intervention or not. CTs are used for preoperative planning for device deployment. Around 24.7% of patients have PAR in 2 years after TAVR (Kodali et al., 2012), and 25% of patients have atrioventricular block (Kapadia et al., 2017; Makki et al., 2017) with current interventional planning approach. The incidence of PPI varies according to the valve design and was previously reported to be significantly lower with the balloon-expandable SAPIEN (Edwards Lifesciences, Irvine, California) than with the self-expanding CoreValve (Medtronic, Minneapolis, and Minnesota). Incidence of AVB with CoreValve implantation could be high up to 41.3% after 2 years (Sondergaard et al., 2016). TAVR is expected to be used in some medium-to-low risk patients in near future. Avoiding AVB and PPI could reduce costs, complications and improve long-term outcome. For patients with high risk for PPI and paravalvular regurgitation, surgical aortic valve replacement may be chosen. However, for those patients with low risk for PPI and paravalvular regurgitation, TAVR will certainly be a vital option. It is extremely challenging to optimize the valve size alone to prevent a complete AVB yet. Thus, we aim to develop a robust and comprehensive prediction model to delineate the predictive factors for AVB.

The higher rate of new PPI with CoreValve is likely due to its stent design and properties that influence the position of the valve frame within the left ventricular outflow tract (LVOT) and the radial force exerted on the conduction system. The mechanical behavior of aortic valve tissue varies with radial force, which is mainly contributed from the altered stent expansion and calcium deposits in different leaflets of AS patients after TAVR (Dasi et al., 2017; Rocatello et al., 2018). If the aortic valve leaflets carry severe and unevenly distributed calcification, excessive stresses could damage the atrioventricular conducting system. If the aortic valve apparatus do not respond to TAVR prosthesis as expected, which is assessed by pre-TAVR CT imaging or 3D echocardiography, TAVR prosthesis might be dislodged, uneven expanding or poor sealing, leading to significant AVB.

Conduction system injury requiring PPI becomes increasingly clinically relevant as TAVR is employed in medium-to-low risk patients, given the potentially increased morbidity of PPM in patients with longer life expectancies and the low incidence of PPM implantation if patients are treated surgically (Steinberg et al., 2012; Judson et al., 2019). Therefore, identification of the patients at high-risk for PPI post TAVR may help guiding appropriate management strategies.

## Materials and Methods

### Patients and Methods

This study presents an integrated approach to accurately simulate aortic tissue behavior for pre- and post-intervention, respectively, for the purpose of early prediction of AVB after TAVR and estimate the optimal size and shape of prosthetic valve. Our hypothesis is that analysis of 3D reconstruction and tissue characteristics of the aortic valve apparatus may identify high-risk patients requiring PPI after TAVR.

A total of 48 patients (28 males, 20 females) with an average age of 79 years, ranging from 71 to 88 years, and average body mass index (BMI) of 29.3 kg/m^2^, ranging from 21.3 to 42.5 kg/m^2^, were included in the study. All patients were implanted with self-expandable CoreValve System (Medtronic, Luxembourg) during TAVR procedure and had been followed for at least 2 years to assess the risk of AVB. All clinical data were collected at Wake Forest Baptist Medical Center. We divided them into two cohorts based on clinical outcome, with cohort A consisted of 28 patients underwent TAVR only (none AVB) and cohort B consisted of 20 patients underwent TAVR failure (significant AVB).

Full phase cardiac CT scan was acquired using a GE Light Speed 64-channel volume computed tomography scanner. A total of 2,500 slices with thickness of 0.625 mm were collected for the whole cardiac cycle. 3-dimensional (3D) reconstruction of systole cardiac geometry was performed with a window width of 950 and −50 Hounsfield units using Mimics software. Aortic root and left ventricle were then identified and separated to form a 3D model. Meshing for the aortic complex model with solid elements (eight-node hexahedral C3D8I and four-node tetrahedral C3D4) was performed using TrueGrid software.

**FIGURE 1:**
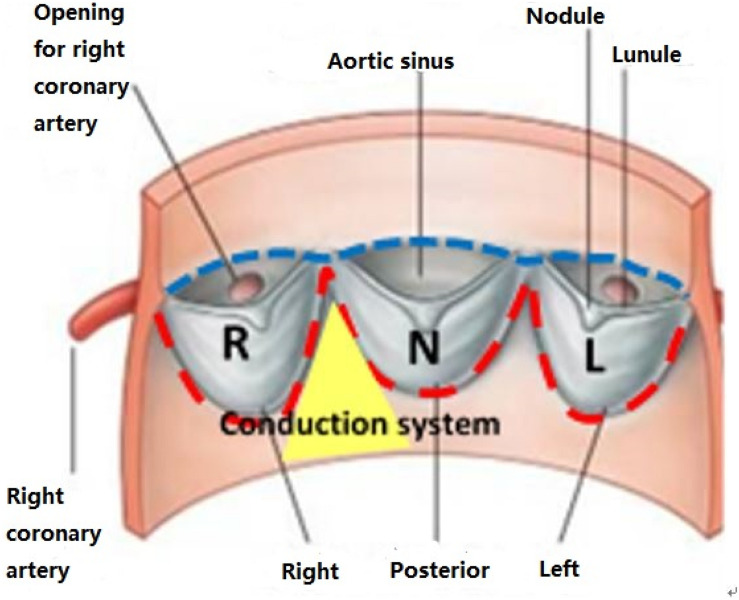
Conduction system zone.

**FIGURE 2:**
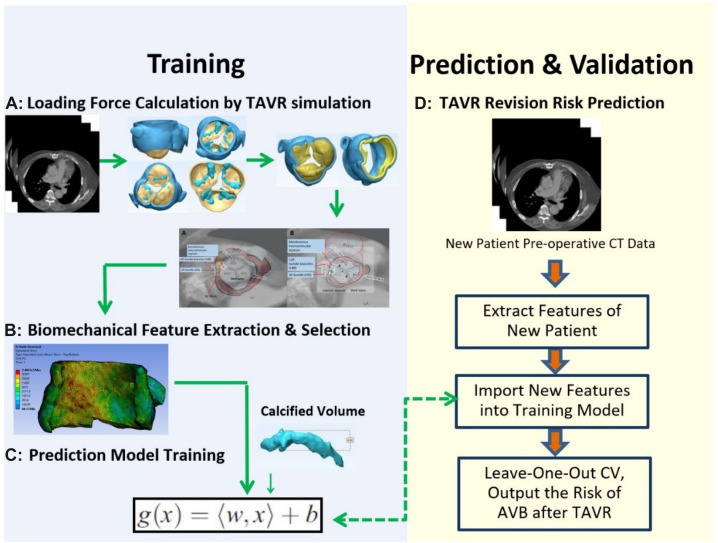
Workflow for AVB prediction.

CT images of 48 patients with TAVR were analyzed using 3D anatomical models of the aortic valve apparatus (segmentation) created by manually thresholding images from 320–800 HU for the separation of the aortic root from the atria and ventricles and 800–1,400 HU for the calcium deposits. Interleaflet triangle region between NCC and RCC was defined as the “conduction system zone” (Figure 1), where cardiac conduction system, AV bundle specifically, entered the ventricular septum. Volumes of calcium deposits located in this area after TAVR were measured. The workflow of the study is shown in Figure 2.

### Feature Extraction

In this study, we analyzed leaflet sectors of the aortic-valvular complex with respect to the need for PPI. Usually, patients requiring PPI had larger right coronary cusp (RCC) or noncoronary coronary cusp (NCC) sector calcification in the region of the leaflets (Dasi et al., 2017; Li and Sun, 2017; Lin et al., 2020; Padala et al., 2021). When a prosthesis was deployed within a severely and asymmetrically calcified leaflet during TAVR, there was a large pressure generated from the calcified leaflets. Severe calcification within left coronary cusp sectors could induce a large stress on the contact zone, while the AV bundle might still be intact. Conversely, patients who underwent PPI had larger RCC and NCC sector calcification in the device-landing zone. The presence of excess calcium may result in extrusion or trauma, with the calcium compressed by the stent frame directly onto the cardiac conduction system in this region. Thus, the specific anatomic distribution of calcification was measured and simulated to predict AVB in this model.

**FIGURE 3:**
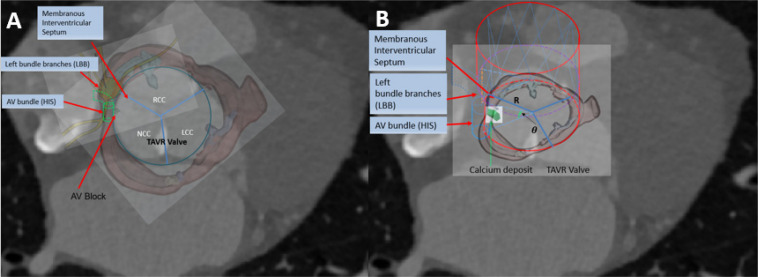
Aortic Tissue with TAVR. **(A)** AVB with TAVR; **(B)** Optimal valve.

We simulated the device-landing of the TAVR to avoid AVB. The basic idea behind the optimization of the valve shape (Figure 3A) is as follows. To reduce the excess force exerted on the conduction system, we attempted to remove the overlapping region on the stent where calcium in contact would extrude the interleaflet triangle region between NCC and RCC (Anderson et al., 2009; Caballero et al., 2019; Spadaccio et al., 2020). It was difficult to accurately determine this region in clinic. We recently proposed an approach (G, Zhang et al., 2017) to measure the overlapping regions caused by the 3D calcium deposit of the valve apparatus within a 90° central angle column of the aorta (Figure 3B). The overlapped region was defined as cylindrical sector with angle θ (Figure 3B). Thus, the optimization of the Valve shape would be the joint optimization of the θ and Valve size*R* (*r*_*size*_).

Volume of calcium deposits in the native aortic valve was measured to evaluate the severity of leaflet calcification. In TAVR, the prosthetic valve deployment was accomplished following the outward expansion of prosthesis within aortic annulus. An expansive force was exerted on the aortic wall. The calcium deposits surrounding the stent would result in excess loading forces against the aortic root. We assumed that all calcium deposits on the native leaflets would adhere to the aortic wall after TAVR and the displacement as well as rotation of each calcium deposits could be calculated accurately.

**FIGURE 4:**
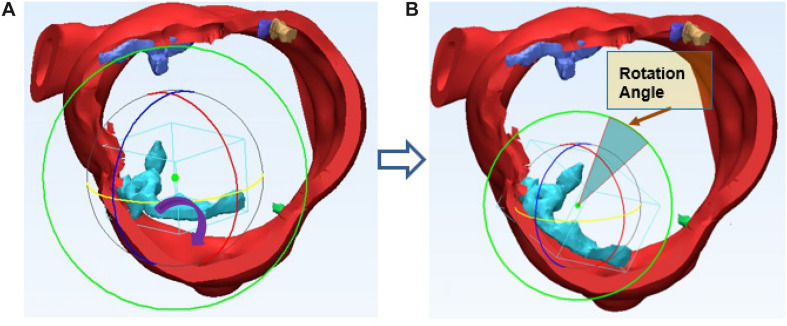
Irregular calcium deposit rotation simulation. **(A)** preoperative; **(B)** postoperative.

Thickness *d* of each calcium deposit was first measured. After 3D reconstruction of aortic root with the calcium deposits, each calcium deposit was measured, max thickness = 6.8 mm, average thickness = 3.2 mm, equivalent cylinder thickness, center of the deposit and aortic root was also determined and marked on the preoperative 3D model. Calcium deposits were assumed to shift along the virtual radial line generated by the center of deposits and end up contacting the aortic wall. In TAVR, the aortic annulus allows for outward expansion (displacement) of prosthetic valve deployment. The prosthetic valve produces an expansive force to outspread aortic annulus. The virtual rotation procedure is assuming the distribution of calcium deposits would realign to the aortic wall of the annulus, each calcium deposit changes with displacement and rotation following virtual stent expansion can be calculated precisely. Position changes in calcium deposits with native aortic valve following balloon-expand/self-expand during TAVR will generate different loading forces against the aortic root. For the irregular calcium deposits, we assumed rotation would also occur. The virtual rotation would make both ends of the calcium deposit contact the aortic wall as shown in (Figure 4).

### Determine Material Property of the Aortic Tissues

Anatomic details of the aortic tissues were incorporated in the mesh data. Material parameters for the tissue including Young’s modulus and Poisson’s ratio were collected from previous studies as shown in Table 1 (Zioupos et al., 1994; Xiong et al., 2010). Aortic tissues were defined as homogeneous and isotropic property. To assign different material properties to FEM mesh data, aortic root and native aortic valve leaflets were segmented and measured. The wall, leaflets, calcium depositions, has been measured as having different material parameters. Since the native leaflets were extruded to and surrounded by the aortic root after TAVR, the overall aortic tissue properties were determined according to the volumetric proportion of aortic root and leaflets. The volume of the two components were measured based on CT data using the Mimics software. The volumetric proportion of aortic root represented by *P*_*root*_ was then calculated. Material parameters in terms of Young’s Modulus were determined by *E* = *E*_*valve*_(1-*P*_*root*_)+*E*_root_*P*_root_. Poisson’s ratio υ was similarly calculated. Using the properties above, tissue behavior was simulated based on Hooke’s Law.

**TABLE 1:** Material parameters of aortic tissue.

	Young’s Modulus [M Pa]	Poisson’s Ratio	Density [kg/m^3^l
Aortic root	2	0.45	2,000
Aortic valve	8	0.45	1,100

The segmented aortic roots with aortic valve and calcified regions were first discretized the 3D object into small elements (called mesh). The mesh data were generated by using TrueGrid (XYZ Scientific Applications, Inc., Livermore, CA, United States). It is composed of 7,820 hexahedral elements and each element contains eight mesh nodes. To limit the number of elements and reduce the computational complexity, FEM calculation was only restricted to the aortic annulus tissue which is the landing zone of the implanted prosthesis represents the most relevant anatomic structure with regard to aortic stenosis. The mesh nodes can be classified into the boundary and free nodes. The boundary nodes are located in the wall surface parts, which would be repositioned when the stent expands. The free nodes are subject to the displacement of boundary nodes. The displacement boundary condition consisting of the displacements of all the boundary nodes, will be simulated by FEM *via* adding a loading force on the internal surface of the aortic wall tissue. The stress modeled by Hooke’s law with respect to these parameters. Here, FEM was implemented by the commercial FEM software ANSYS 12.0 (ANSYS Inc., PA, United States). The mesh-independence and Linear elastic solver-independence are approved in ANSYS, following convergence, additional mesh refinement does not affect results, and Linear elastic solvers is enough for characterizing the tissue behavior at this scale.

### Formulation of Stresses According to Loading Force and Tissue Properties

**FIGURE 5:**
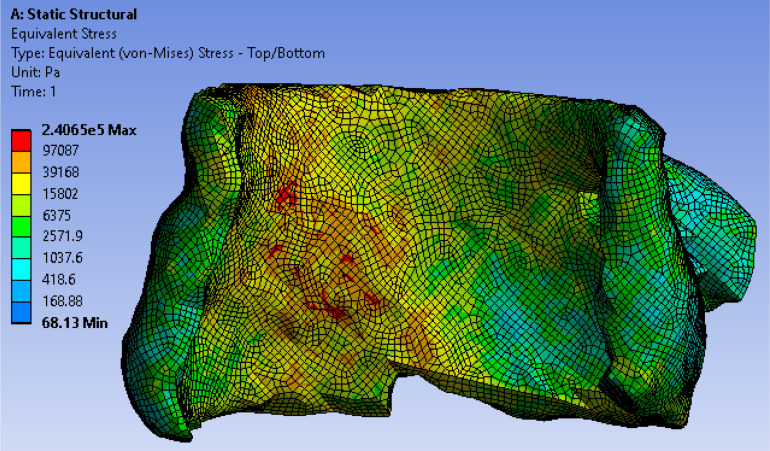
Stress distribution in aortic tissue.

In TAVR, the outward expansion of prosthetic valve would produce an expansive force to the surrounding aortic annulus. To accurately simulate the virtual deployment before TAVR, stress distribution was calculated according to the loading force and tissue properties. We assumed the stent was fully unfolded, thus the loading force F could be calculated by geometric relationship as *F* = *K*⋅(*R*−*C*_annu_/2π + *d*), where *K* represented the stiffness of aortic wall, *R* represented the valve size, which is slightly larger than the native aortic, and *d* represented the thickness of calcium. Valve size *R* and the angle θ of valve cylindrical sector (Figure 3B) will be optimized in the force calculation workbench and prediction model to avoid excessive stress prior to the surgery to ensure the successful operation effectiveness. We extracted stresses as biomechanical properties from 7,820 hexahedral elements in FEM (which can fit the aortic tissue well with less than 2% error) by simulating a virtual prosthetic valve deployment in ANSYS 12.0. Here we adopted hexahedron to construct 3D mesh because it has advantages in convergence, error estimation and computation time. In FEM model, the stress of each grid node was affected by the components of aortic annulus. In order to obtain the stress distribution, we first simulated the valvular stress in response to the loading force. Afterward, the stress vector σ_**i**_ was stacked together and employed as the feature of the **i**th patient. We denoted $\sigma_{i}=\sigma \,\left(F_{i},E,\upsilon ,A_{i}^{\left(n\right)},\theta_{i}\right)$, where, *F_i* was the loading force of the **i**th patient, $A_{i}^{\left(n\right)}$ denoted the cross-sectional area for the mesh node *n*, θ_*i*_ denoted prosthetic valve shape i.e., the angle of cylindrical sector, and σ(⋅) was the function representing the stress modeled by Hooke’s law (Fung, 2004) with respect to these parameters. One example for the stress distribution was shown in Figure 5. All finite element analysis was performed on ANSYS 12.0 (ANSYS Inc., PA, United States).

### Modeling the Relationships Between Clinical Outcome (AVB), Clinical Factors, and Biomechanical Properties

Important aortic complications i.e., AVB after TAVR are strongly related to the biomechanical properties such as stress induced by the prosthetic valve and selected clinical factors. We extracted the electronic medical records as clinical factors from EPIC system. Gender, BMI, systolic pressure, diastolic pressure, left ventricular ejection fraction (LVEF), calcium volume, calcium location, distribution, diabetes mellitus, history of atrial fibrillation, history of stroke and intracerebral bleeding were selected as important clinical factors for the prediction of AVB. In our previous study (Zhang et al., 2016a), SVR was employed to model such relationship because this model can efficiently model any nonlinear functions with small sample size. We denoted biomechanical features as $\sigma_{i}=\sigma \,\left(F_{i},E,\upsilon ,A_{i}^{\left(n\right)},\theta_{i}\right)$, clinical factor as calcified volume as *V_i*, and the clinical outcome AVB as $A\,V\,B_{i}=g\,\left[\sigma \,\left(F_{i},E,\upsilon ,A_{i}^{\left(1\right)},\theta_{i}\right),\ldots ,\sigma \,\left(F_{i},E,\upsilon ,A_{i}^{\left(N\right)},\theta_{i}\right),V_{i},W\right]$, where N was the total number of mesh nodes in interleaflet triangle area between NCC and RCC, *g* was the support vector regression model *g*(*x*) = < *w*,*x* > *b* with a input vector *x*, and *W* was the parameters (the coefficients of the variables in g) to be determined by minimizing the objective function as W*=a⁢r⁢g⁢m⁢i⁢nW⁢||A⁢V⁢Bi⁢[c⁢p⁢s⁢b⁢r⁢e⁢a⁢k]-g⁢[σ⁢(Fi,E,υ,Ai(1),θi),…,σ⁢(Fi,E,υ,Ai(N),θi),Vi,W]||L⁢2 which was calculated from the loading force *F_i*, the calcified volume *V_i*, material parameters*E*,υ, cross-sectional area$A_{i}^{\left(n\right)}$, prosthetic valve cylindrical sector with angleθ (we set θ = 0° in training data, which represented the complete valve), and the regularization parameter λ> 0. The objective function was optimized by dynamic programming approach (Kishimoto et al., 1980; Kim, 1993; Powell, 2011).

## Experiments and Results

### Clinical Outcome Prediction and the Optimal Valve Size Estimation

When a new patient comes to hospital, the degree of AVB can be observed by echo and ECG preoperatively. *W*^∗^is known from Training step. *E*,υ,*V*,*A*^(1)^,..,*A*^(*N*)^ can be obtained from CT data by FEM for all patients. The optimal valve loading force *F* and the angleθ for this patient can be estimated as below:

(F*,θ*)=a⁢r⁢g⁢m⁢i⁢nF,θ∥R-g[σ(F,E,υ,A(1),θ),…,σ(F,E,υ,A(N),θ),V,W*]∥

Thus, we can get the optimal angleθ of valve cylindrical sector and the optimal loading force. They can be used to help in choosing optimal size *R viaF* = *K*⋅(*R*−*C*_annu_/2π + *d*)in the force calculation workbench to avoid excessive stress prior to the surgery, and to ensure the successful operation effectiveness. The stiffness of aortic wall was represented as K and was previously reported through performing linear regression on force-displacement data (Fung, 2004). The radius of AV bundle was ranged from 0.4–0.8 mm with 95% confidence interval [CI]; *p* < 0.05. Here we chose 0.6 mm for calculation.

### Feature Selection

To identify the most relevant features with a high degree of discrimination between AVB and none AVB, we used our DX score feature selection method. The effectiveness of DX score feature selection method was confirmed in our study. Here, we selected the top 16 features as vector $\text{[}x_{i}^{1},\cdots ,x_{i}^{16}]{}^{\text{T}}$ with 92.1% impact percentage of all features that affect the outcome, for the *i*th patient, *i* 1,,N. High/excessive stresses exerted on the contact zone can injure the atrioventricular conducting system. Our model can also offer a better insight in the mechanism of AVB with dysfunction of AV bundle and valve leaflets. For example, among biomechanical features${\left\{x^{\text{i}}\right\}}_{\text{i}=1,}^{16}$ related coefficients ${\left\{{\text{w}}^{\text{i}}\right\}}_{\text{i}=1,}^{16}$ the {w^4^,w^7^,w^12^,w^15^} were the biggest numbers that contribute mostly for the prediction model. These four coefficients represented the biomechanical features (excessive stresses) in the contact area between tissues and stent, which can cause the functional blockage of AV bundle, leading to TAVR complications.

### Sensitivity Analysis

**FIGURE 6:**
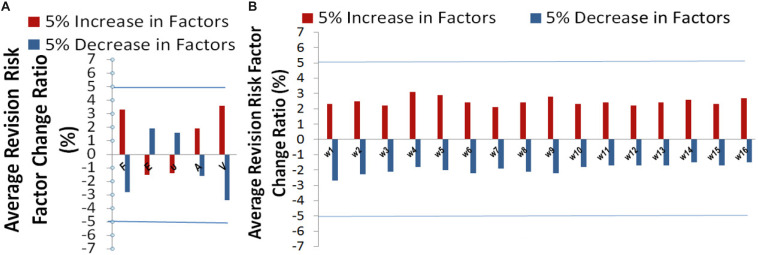
Sensitivity analysis for **(A)** the variables related parameters, and **(B)** model parameters: coefficients ${\left\{w^{i}\right\}}_{i=1}^{15}$, and *w*^16^ (corresponding to calcified volume).

Sensitivity analysis (Zhang et al., 2016a) was performed to explore the model output variation upon perturbation of variables (Zhang et al., 2016b) such as loading force *F*, Young’s modules *E*, Poisson’s ratio υ, mesh related cross section area *A*, calcified volume *V*, and parameters such as 16 coefficients ${\left\{w^{i}\right\}}_{i=1}^{16}$ which represented the important features *via* DX score (Zhang et al., 2019). A perturbation over a range of 5% was performed on all factors. Overall, the output variance of our model was bounded by 5%, which proved the stableness of our model. The effectiveness of each factor in our model was shown in Figure 6. Risk factor of AVB was quite insensitive to the perturbation of material related parameters such as Young’s modulus and Poisson’s ratio (1.5–2.0% under perturbation), while it was more sensitive to the perturbation of biomechanical features and clinical factors of calcified volume (2.7–3.8% under perturbation). Our model can potentially underlie the mechanisms of TAVR outcomes.

The accuracy of our model was assessed using leave-one-out cross validation (LOO-CV). One of the patients was selected as testing sample each time, while the remaining ones served as training sample. We kept repeating this procedure until each patient had been used as a testing sample. 2 years clinical data of ECG study post procedurally was used as ground truth and compared with the predicted results. Cases 1–28 in Group A (43% females) underwent TAVR without AVB, and Cases 29–48 in Group B (40% females) had significant AVB. Table 2 represents the performance of our biomechanical property-based machine learning approach. The average AVB risk prediction accuracy was 83.33% in the range from 0.8–0.85 with 95% CI for all cases; specifically, 85.71% for Group A (no AVB), and 80.0% for Group B (undergoing AVB after the TAVR). Case #4, Case #13, Case #21, and #23 failed because of prosthetic unstable due to much calcification at first, but gradually fit the aortic root. These cases required a novel stabilized valve. Case # 34, # 39, # 41, and # 44 failed because pre slight AVB existed. The TAVR procedure may aggravate the disease during the valve deployment. Our data consist of 42% female and 58% male patients. We applied our approach to them and didn’t see any significant difference between males and females in risk prediction.

**TABLE 2:** Prediction performance on two groups.

No.	True type for replacement	Calcified volume (mm^3^)	Predicted results (O-TAVR without AVB. 1 = complications with AVB) percentage (0–1)	Applied valve size (mm)	Optimal valve size (mm)	Valve vacant angie (°)
1	TAVR	403	0.235	26	26	0
2	TAVE	425	0. 163	29	29	0
3	TAVR	386	0. 348	31	30	0
4	TAVR	445	0.651 (AVB)	23	24	6
5	TAVR	512	0.233	26	26	0
6	TAVR	683	0.315	23	24	0
7	TAVR	461	0.388	26	26	0
8	TAVR	442	0. 191	26	26	0
9	TAVR	408	0. 253	29	29	0
10	TAVR	392	0.411	29	29	0
11	TAVR	354	0.42	26	27	0
12	TAVR	362	0. 286	23	23	0
13	TAVR	401	0.756 (AVB)	26	27	5
14	TAVR	385	0.341	26	26	0
15	TAVR	477	0. 295		28	0
16	TAVR	396	0. 196	23	in	0
17	TAVR	362	0. 283	23	23	0
18	TAVR	318	0. 322	26	25	0
19	TAVR	452	0.431	29	28	4
20	TAVR	557	0. 268		23	0
21	TAVR	460	0.624 (AVB)	23	23	6
22	TAVR	381	0. 375	26	26	0
23	TAVR	446	0.685 (AVB)	26	25	5
24	TAVR	419	0. 234	23	23	0
25	TAVR	396	0. 265	26	26	0
26	TAVR	501	0.358	29	28	5
27	TAVR	384	0.287	26	25	0
28	TAVR	396	0. 327	26	25	0
29	TAVR+PPM	516	0. 902	26	24	8
30	TAVR+PPM	532	0. 845	23	25	10
31	TAVR+PPM	490	0. 736	23	25	6
32	TAVR+PPM	425	0.617	26	28	7
33	TAVR+PPM	342	0.839	26	25	10
34	TAVR+PPM	556	0. 393 (no AVB)	29	27	0
35	1AVR+PFM	432	0.926	26	27	9
36	TAVR+PPM	323	0.827	26	28	12
37	TAVR+PPM	472	0. 738	29	27	8
38	TAVR+PPM	436	0.868	31	30	7
39	TAVR+PPM	502	0. 277 (no AVB)	26	27	0
40	TAVR+PPM	487	0.845	23	25	6
41	TAVR+PPM	496	0. 326 (no AVB)	29	31	0
42	TAVR+PPM	533	0.764	26	25	9
43	TAVR+PPM	412	0. 923	26	26	5
44	TAVR+PPM	477	0. 287 (no AVB)	29	27	0
45	TAVR+PPM	507	0.831	31	30	5
46	TAVR+PPM	405	0.846	26	26	6
47	TAVR+PPM	387	0.798	29	28	7
48	TAVR+PPM	452	0.824	26	27	5

## Discussion

Our studies (Table 3) showed that SVR significantly outperforms other risk prediction models such as logistic regression, decision trees, and neural networks when sample size was small and data have nonlinear property. The receiver operating characteristic (ROC) curve was employed for performance comparison. The area under the curve (AUC) of prediction was 0.92, indicating our VBPR model outperforms the logistic regression (AUC = 0.86), decision trees (AUC = 0.84), and neural networks (AUC = 0.89). We then compared the performance of AVB prediction with different feature groups: clinical factors only, biomechanical factors only, and the combined factors (Table 4). The results showed that just clinical factors failed to predict the TAVR failure, and our approach VBPR with the combined factors outperformed the other risk prediction models (based on biomechanical features only AUC = 0.88, and based on clinical factors only AUC = 0.75).

**TABLE 3:** Performance comparison with the existing standard machine learning methods.

Machine learning methods	Multiobjective optimization by support vector regression (SVR) i.e., VBPR	Logistic regression	Decision trees	Neural networks
Accuracy *(%}*	83.33	73.62	73.7	76.8
AUC	0.92	0.86	0.84	0.89

**TABLE 4:** Performance comparison with different factors.

Methods	Biomechanical features Methods combined with clinical Factors	Biomechanical features only	Clinical Factors only
Accuracy (%)	83.33	77.5	63.8
AUC	0.92	0.88	0.75

We conducted LOO-CV on the estimated valve size (Table 2), which shows 85.5% accuracy with AUC as 0.90 for valve size, and 83% accuracy with AUC as 0.86 for valve shape based on the toleration error within 2 mm on size and 6° on shape. In brief, the predicted valve size and shape were highly consistent with the actual size and shape in Group A. In group B, our approach predicted the optimal valve size and shape for those patients with TAVR failure. Since those patients underwent AVB, we then conducted clinical validation based on the estimated valve size and shape.

We collected postoperative CT data of 20 patients for validating the Calcium Deposits Offset simulation. We compared the displacement and rotation of the calcium between simulation and post CT. The results showed an exceptional agreement in TAVR patients between the planned and postoperative outcomes.

A significant difference of biomechanical features between two cohorts after TAVR simulation was observed in our study, which indicate the necessity of optimizing the implanted valve size and shape before intervention. It is worth noting that our optimized prosthetic is only an approximation and it could be more precise on the patient-specific level. Ideally, 3D imaging model from CT scans may be performed at the prosthetic valve stent deployment phase such that an accurate estimation of the aortic root geometry at 0–20 mmHg can be obtained. The cylindrical sector with angle θ estimated in our model can eliminate the excessive stresses close to the AV bundle, which can damage the atrioventricular conducting system. Thus, this model can avoid AVB and PPI to reduce costs, complications and improve long-term outcome.

Transcatheter aortic valve replacement was simulated based on the patient-specific FE model reconstructed from preprocedural CT data in our study. A higher mechanical response of aortic root was observed in patients with AVB than those without AVB. In surgical aortic valve replacement, the calcified leaflets are removed, while in TAVR, the calcification deposits were left within the annulus and extruded to the aortic wall, which might result in AV bundle injury.

Besides, the aortic wall residual stress was taken into account in this model. The aortic valve was heavily calcified for AS patients. The residual stress caused by the calcification on the aortic wall was calculated in this study, which made the aortic root more compliant during and after TAVR simulation. In summary, this model performed well in predicting the risk of AVB for the patients who need TAVR. This model can also estimate the optimal valve size and shape. This optimization may eliminate the excessive stresses to keep the normal function of both AV bundle and valve leaflets, leading to a favorable clinical outcome. The combination of biomechanical properties and machine learning method substantially improved prediction of surgical results. The prediction error in some cases may be caused by material nonlinearity.

The limitations of this study are that there is very small numbers and we do not have the capability of accounting for potential factors other than biomechanical. Machine Learning requires massive data sets to train on, and these should be inclusive and of good quality. This exploratory study may suggest that the proposed methodology may be useful but its clinical efficacy shall be assessed. The precision of the ML method is based on the quality of its inputs and biases in the data sets provided can lead to inexact outcomes. If the training data is not well balanced, the method will amplify the discrimination and bias that lies in the data set. The most ideal way to mitigate such risks is by collecting data from multiple random sources. Therefore, further validation of the proposed methodology on a large cohort shall be conducted to assess the reliability of this preliminary work. And, when conducting larger trials to validate the results of this approach, it shall be ensured enough data heterogeneity.

Common cardiac risk factors including diabetes mellitus, hypertension and congestive heart failure have been associated with the development of left bundle branch block (LBBB) and pre-existing AV block, those patients are excluded in this study. Biomechanical simulation is time-consuming and labor-intensive and largely dependent on the aortic tissue properties, thus, it is important to develop a novel biomechanical property-based machine learning approach to model the relationship between biomechanical properties and risk of incidence of AVB after TAVR.

Preoperative demographic data showed no difference between two groups with respect to age, preoperative rhythm, and gender (p > 0.05). 3D reconstruction and tissue characterization of the aortic valve apparatus could identify the majority of the patients with severe aortic stenosis at high-risk for PPI after TAVR. With development of an automatic algorithm, this method may potentially become part of comprehensive risk evaluation prior to TAVR and reduce PPI burden as well as cost. The requirement of PPI is a rare complication of isolated TAVR. In our study, annular calcification closed to the conduction system zone is found to be associated with increased risk of PPI after TAVR.

## Data Availability Statement

The original contributions presented in the study are included in the article/supplementary material, further inquiries can be directed to the corresponding author/s.

## Ethics Statement

The retrospective study involving human participants was reviewed and approved by the Medical Research Ethics Committee of Wake Forest Hospital. The written informed consent of patients/participants were waived in this study.

## Author Contributions

RL: data collection. GZ: data analysis and writing of the manuscript. RL and MP: data interpretation. XZ: research conception. GZ and XZ: critical revision of the manuscript. All authors reviewed and approved the manuscript.

## Conflict of Interest

The authors declare that the research was conducted in the absence of any commercial or financial relationships that could be construed as a potential conflict of interest.
